# Learner experiences of safety at public high schools in three South African townships: Baseline findings from the National School Safety Framework learner surveys

**DOI:** 10.12688/gatesopenres.13328.2

**Published:** 2023-10-26

**Authors:** Alison Kutywayo, Khuthala Mabetha, Nicolette P. Naidoo, Tshepo Mahuma, Paseka Njobe, Ronelle Hlongwane, Saiqa Mullick

**Affiliations:** 1 Wits RHI, Faculty of Health Sciences, University of the Witwatersrand, Johannesburg, Gauteng Province, South Africa; 2 School Safety Directorate, National Department Of Basic Education, Pretoria, South Africa

**Keywords:** school safety, violence prevention, National School Safety Framework (NSSF), South African schools

## Abstract

**Background:**

Despite progressive policies and frameworks on school safety by the Department of Basic Education, safety remains a concern in South African schools.

**Methods:**

A cross-sectional descriptive design was employed using the National School Safety Framework (NSSF) 152-question learner survey, exploring perceptions and experiences pertaining to eight safety domains: dangerous objects, drugs and alcohol, bullying, verbal abuse, physical violence, discrimination, sexual violence, and journey to and from school. Grade 9-11 learners from 15 government-funded high schools in the Girls Achieve Power trial in Khayelitsha, Soweto, and Thembisa townships were surveyed (March 2018 - April 2019), sampling 10% of the school population. Data analysis included Principal Component Analysis (PCA), reducing correlated variables into fewer questions, then analysis on a scree plot by calculating eigenvalues; repeated PCA with those that had a minimum eigenvalue of 1 and Cronbach Alpha test for internal reliability. Eleven composite variables were included in the final analysis.

**Results:**

In total, 1034 learners completed the NSSF learner survey; 52.9% were female and the mean age was 16 years (SD=1.36). Results show statistically significant associations between four of the 11 composite variables in relation to sex. Over half (55%) of males have experienced peer provocation and relational aggression (p<0.001). Fifty-eight percent of females reported feeling unsafe on their way to and from school (p<0.003). Over half of males reported that their school was not effective in enforcing discipline (p=0.002) while 58% of females noted they could comfortably report any form of experienced or witnessed violence at school, to their educators (p<0.000).

**Conclusions:**

Violence continues to be a concern in South African schools. Interventions should work across the ecological model to effectively prevent and reduce violence at school and community levels. Strengthened NSSF implementation is critical to achieving this. We recommend NSSF learner survey adaptations to increase utility and implementation.

## Introduction

Violence during childhood and adolescence can have lifelong adverse health, social, educational and economic consequences ^1– 3^. In particular it can increase vulnerability to HIV acquisition ^4^ due to increased likelihood to engage in risky sexual behaviours, cause poor academic performance ^2, 5, 6^, and can lead to higher levels of depression and suicide ideation ^7^. Substance use also is a public health concern, as a predictor of violence ^4^. While children who are not exposed to violence can still be violent, children who are constantly exposed to violence display violent behaviours more often than those who are not ^8^. Other characteristics can lead to some children being more likely to be bullied than others. 

Schools should be a conducive teaching and enabling environment for both learners and educators; however, violence in South African (SA) schools continues to be a problem, despite progressive school safety policies and implementation frameworks by the National Department of Basic Education (DBE) ^9– 11^. Schools have become unsafe places, where learners are at risk of experiencing violence and in some cases death during school hours, in after-school programs, and on their way to and from school ^2, 12, 13^. Perpetration of violence and victimization in schools has two dimensions: 1) learner-learner violence where learners may bully, harass, rob or assault each other of their valuables; and 2) educator-learner or learner-educator violence ^14^.

Multiple studies and media coverage over the last decade have highlighted the severity of school violence ^2, 9, 15– 18^. According to these studies, bullying stands out as a common act of violence in SA, where in many cases learners are perpetrators of violence, with both, learners and educators becoming victims ^19^. In the 2019 Organisation for Economic Co-operation and Development (OECD) Teaching and Learning International Survey, 34% of SA principals report that acts of intimidation or bullying among their learners occur at least weekly in their school, more than double the OECD average ^20^. Linked to bullying, data from the 2016 SA school violence against children (VAC) survey with 15 – 17 year olds, showed that 82.0% reported experiencing some form of victimisation whether criminal victimisation or exposure to family or community violence ^21^. Another study indicated that 22% of learners aged between 12 –18 years across SA having experienced some form of violence while at school in the past year ^2^. When it comes to sexual harassment, the same VAC study showed that over a third of young people had experienced some form of sexual abuse at some point in their lives and almost 10% had been made to do sexual acts against their will by a peer ^21^. A qualitative study with 13 – 17-year-old SA learners found that both boys and girls are perpetrators and victims of sexual violence in schools ^22^. Research reveals that there is poor management of violence in SA schools, with many educators and learners fearing retribution from the perpetrator ^22^. This suggests that reporting the incidence of violence to educators is generally ineffective ^22^, resulting in an unending cycle of violence. Regarding drug and alcohol use, principals report weekly incidents related to the use or possession of drugs and/or alcohol at school (South Africa 27%; OECD average 1%) as well as vandalism and theft (South Africa 21%; OECD average 3%), which are comparatively very infrequent in other countries ^20^. 

With regards to violence perpetrated by educators against learners, Burton ^15^ revealed that school principal management reports show that 50% of educators have verbally abused learners, and 25% of educators have physically abused learners. Despite being banned through the National Education Policy Act of 1996, corporal punishment, *defined as any kind of violent action inflicted on children by educators or school administrators as punishment for disciplinary purposes*, is still used as a common form of discipline in many schools ^22, 23^. Provincial rates of corporal punishment vary between 22.4% in Gauteng to 73.7% in Kwa Zulu Natal ^2^.

Learner and educator safety is the mandate of the DBE, led by the School Safety Directorate. In response to an accumulation of safety concerns for both learners and educators, the DBE, the Centre for Justice and Crime Prevention and UNICEF South Africa introduced and launched the National School Safety Framework (NSSF) in 2015, as an update to the Hlayiseka Early Warning System ^24^. The NSSF is located within a range of international and national laws and policies that recognise the safety of learners and educators as a prerequisite for quality learning and teaching at school ^25^. The NSSF provides an important instrument through which minimum standards for safety at school can be established, implemented and monitored, and for which schools, districts and provinces can be held accountable ^25^. The four strategies of the NSSF are as follows:

- Schools should have effective strategies that aim to prevent any issues that may hinder safety in schools.
- Schools should remain alert on what transpires in the school premises by implementing data collection tools that are related to the NSSF.
- Schools should be action-oriented through the implementation of effective school policies and management that focus on safety.
- Schools should foster the development of good relationships between all members of the school body and referring learners to services that focus on violence perpetration and victimisation ^25^

Late in 2015, the Wits RHI received funding from the Bill and Melinda Gates Foundation to conduct the Girls Achieve Power (GAP Year) trial. As a cluster randomised control trial across 26 schools in Gauteng and Western Cape, GAP Year sought to test the effectiveness of a four-pronged ecological intervention: a sports-based after-school asset-building component, a parent engagement component, linkage to care component and a school safety component ^26^. The school safety component included supporting the DBE to implement the NSSF. GAP Year schools were assessed, in partnership with DBE, using the NSSF implementation framework to determine the state of violence prevention, management, and reporting, seeking to create an enabling environment for adolescents by transforming schools into hubs of safety and support. The primary outcomes of GAP Year were to reduce school dropout of adolescent girls between grades 8–10 and increase reporting of gender-based violence (GBV). Complimenting these outcomes, the four-pronged intervention sought to improve adolescent girls’ agency and safety while shifting gender attitudes and encouraging positive behavioural change among adolescent boys.

This paper provides the results of the baseline learner survey, one component of NSSF implementation. It also contributes to the ongoing routine monitoring of the NSSF and the little, yet growing, evidence base of violence and safety at SA public schools: the last school safety survey was in 2016 ^21^ by Artz *et al.,* and before that, 2012 ^2^, by Burton *et al*., on behalf of the DBE. This manuscript also seeks to inform the development and design of effective interventions and policies to address the prevailing concerns of safety and violence in schools and provides recommendations for the use of and analysis of the NSSF learner survey tool.

## Methods

### Design and setting

A cross sectional descriptive design was employed using the NSSF 152-question learner survey, designed by the Centre for Justice and Crime Prevention with the DBE and UNICEF South Africa (a copy of the survey instrument is available in *Extended data* ^27^). NSSF implementation and monitoring is part of routine DBE programming and is therefore not a research study. In line with this, the methodology and tools were predefined. Recruitment and data collection for the baseline survey was conducted in the Khayelitsha, Soweto and Thembisa townships, SA between March 2018 – April 2019. Khayelitsha is situated in the Cape Town metropole, in the Western Cape ^28^. Much of the population are Black African and 37.2% are under 19 years of age ^28^. Soweto, located to the south west of Johannesburg in Gauteng Province, has a population with 32.6% under 19 years ^29^. It comprises 84.2% formal dwellings and 6357 persons per km ^2 29^. Thembisa, also located in Gauteng province, has a population with 29.2% under 19 years, 72.5% formal dwellings and 10 820 persons per km ^2^ ^30^. These sites and schools (26) were invited to participate as they were part of the ongoing GAP Year trial. The GAP Year school selection inclusion criteria were as follows: mixed sex government funded high schools in Khayelitsha, Soweto and Thembisa; in quintiles 1–3 ^1^, which had not been exposed to any asset building interventions in the past six months. Supported by the School Safety Directorate in the DBE as well as the district DBE stakeholders and circuit managers, the team had various engagements with school principals of all 26 GAP Year schools to explain the NSSF survey, the benefits of the data collected and the whole NSSF implementation process. Following these engagements, only 15 of the 26 schools agreed to participate, whilst others had competing priorities at the time of the study.

### Population and sampling technique

Grade 9–11 learners, ranging from 13 – 19 years, from 15 government-funded high schools participating in the GAP Year trial were surveyed, using the predefined NSSF methodology ^24^ (page 65); specifically, classes were randomly selected from all the classes in the participating grades, ensuring that at least 1 class per grade were selected, per school. All participants in that class were given the opportunity to participate, irrespective of sex, race or age. As per DBE directive ^24^, the survey excluded Grade 8 and grade 12 learners; grade 8 learners were new and less familiar to the school environment while grade 12 learners would have left the school when endline assessments were conducted in the following year.

### Instrument and data collection

An existing DBE-approved learner survey ^24^ (pages 70 – 77), consisting of 152 questions, was used to collect data on experiences and perceptions of violence and safety within the ongoing GAP Year trial. The tool had Likert scale responses, and comprised of 8 sections, covering the following themes: (1) Dangerous objects; (2) Drugs and Alcohol; (3) Bullying; (4) Verbal abuse; (5) Physical violence; (6) Discrimination; (7) Sexual violence and (8) the journey to and from school. This survey sought to elicit learners’ experiences and perceptions of school safety and violence. The GAP Year research team led the recruitment of participants and went from classroom to classroom, clearly explaining the survey purpose to learners and invited them to participate. Learners self-completed the survey in their classroom, during school time, as agreed with the principal and class educator ^24^. The survey took approximately 30 minutes for learners to complete and no personally identifying information was collected on the survey tool, potentially reducing bias. While the survey was being completed, the research team sought to ensure that learners were not looking at each other’s answers. The team also circulated around the class to ensure that the questions were correctly understood.

### Ethics statement

The NSSF is a nationally mandated program of DBE, and these data were collected on behalf of the DBE. The learner survey is part of routine DBE programming and not a research study, and under DBE’s jurisdiction does not require ethical approval. In line with this, the NSSF methodology was carefully applied to ensure learner safety.

The GAP Year trial did receive ethical approval from the Human Research Ethics Committee (HREC) based at the University of the Witwatersrand (#M160940). 

Parental consent was not sought from learners participating in the NSSF learner survey for the following reasons: the schools wherein learners are enrolled serve *in loco parentis* and therefore provide guardianship for learners when participating in a myriad of DBE organised programmes with its partners. This responsibility, known as the ‘ *in loco parentis*’ principle, tasks educators to act in the place of a parent by carrying out legal responsibilities and functions in line with the Fundamental Rights of Children in the Constitution of the Republic of South Africa (CRSA) of 1996. Educators have a duty-of-care and supervision to learners equal to the task as expected of parent(s); taking responsibility for the emotional, psychological and physical well-being of the learners to ensure there is no foreseeable risk of injury to the child.

No written informed consent was sought from learners, however the nature and purpose of the completion of the learner surveys were clearly explained to learners before participation and their assent was provided. Learners were provided with the aims of the study, guaranteed confidentiality, provided details on who will have access to the learners’ information, indicated that the survey is voluntary, provided information on the storage of data, and the dissemination of the findings. We did not collect the number who refused to participate but this could impact the study results as it may not be representative. Willing learners completed the survey at their desk in the classroom and the surveys were conducted anonymously: learner names and other personal information was not collected.

Due to the sensitive nature of the data collection tool, the GAP Year distress protocol was implemented with a social worker available to provide support. Learners who indicated distress during survey completion, were noted by the team and referred to the GAP Year social worker for further support and referrals. Willing learners were informed of their right to withdraw from the survey at any time if they felt the need to do so. It was explained to learners that they cannot pass or fail the survey, as there are no ‘’correct’’ answers. Withdrawal or non-participation from the survey did not affect the learners in any way.

### Data analysis

The main outcome variables were experiences of violence and safety. A four-stepped process was employed in the analysis of data.

Firstly, given the presence of multi-dimensional data, Principal Component Analysis (PCA) was employed as a dimensionality reduction method, reducing large datasets into smaller uncorrelated variables known as “principal components”, whilst maintaining the variance explained by each component ^31^. PCA assisted in reducing 152 questions that were highly correlated, into fewer questions, capturing the strong data patterns that represents the data well. To achieve this, eigenvectors accompanied by their associated eigenvalues (number that indicates how much variance is explained in the data) were first computed. PCA criteria is that components with an eigenvalue >1 should be retained for further analysis, providing more information on the questions to be kept, creating the final composite variables ^32^.

Secondly, all the components obtained in each of the themes were analysed using a scree plot to select the number of relevant components to be considered in further PCA analysis. These were then further determined by identifying variables with the highest correlation and then calculating eigenvalues based on the correlation matrix.

Thirdly, PCA was then performed again limited now to components that had a minimum eigenvalue of 1, using the Orthogonal (Varimax) Rotation method which assisted in clarifying the relationship between the variables by placing them under the relevant components. The Varimax Rotation Method helped minimise the complexity of the factor loadings by isolating factors that had eigenvalues >1.0 and loading them into relevant items with accompanying total variances explained.

Lastly, to test for internal reliability, the Cronbach Alpha’s test was performed. A reliability measure of 0.6-0.7 or above is considered acceptable as it has been largely deemed that a higher alpha value translates into higher reliability ^33– 36^. Based on this, item loadings that had a Cronbach Alpha score of 0.7 were retained in the PCA and used in creating the final 11 composite variables, measuring “violence and safety”. The 11 composite variables were as follows and included in the final analysis:

(1) Experience of peer provocation and relational aggression (Defined as being injured by a dangerous object after being bullied, because of insults, swearing or hate speech, or because someone said something bad about my mother, father, or people important to me, to protect someone else who was being hurt in some way or because someone was trying to hurt me in some way). (measures physical violence)
(2) Experience of peer violence perpetration and victimisation (defined as having been hit, kicked, pinched or punched by a learner or having hit, kicked, pinched or punched a learner) (measures physical violence)
(3) Peer-perpetrated sexual harassment (measures sexual violence)
(4) Perception of feeling unsafe to and from school (measures feelings of unsafety)
(5) Experience of identity-based bullying (measures bullying and verbal abuse)
(6) Experienced verbal abuse from a peer or educator (measures bullying and verbal abuse)
(7) Exposure to illicit drugs on school property (measures exposure to drugs and alcohol)
(8) Enforcement of discipline at school (measures effectiveness of school management)
(9) Ability to comfortably report any form of violence to educators (measures effectiveness of school management)
(10) Presence of code of conduct pertaining to various forms of violence at school (measures effectiveness of school policies)
(11) Exposure to life skills lessons at school (measures effectiveness of school policies)

Appendix 1  outlines the set of questions combined to create outcomes based on Cronbach Alpha score (available in *Extended data*). The responses to each experience of violence were coded as (1) “Yes” for learners who had experienced any form of violence and (0) “No” for learners who had not experienced any form of violence. Descriptive analysis was then used to show the frequency distributions of each of the composite variables. A chi-square test of association (χ ^2^) was then used to test for an association between the 11 composite variables and sex. A p-value <0.05 was considered statistically significant. Stata version 15 ^37^ was used for all data management and analysis undertaken. Missing values were excluded to yield results only for those who had responded to all the key variables.

## Results

A total of one thousand and thirty-four (1034) Grade 9 to 11 learners completed the NSSF learner survey ( Table 1 and Table 2). Overall, 52.9% (n=547) were female and the majority were aged between 16-18 years (58.2%, n=602). The mean age was 16 years (SD=1.36), and all learners were African. A description of learner demographics by sex are found in Table 1. Most participants were from Soweto (47.3%) and were enrolled from Grade 9 (37.1%). Over half of the participants (52.9%, n=547) were female and most females majority were aged between 13-15 years (61%). Of those aged >=19 years, 74% were males. The full, deidentified survey responses are available in *Underlying data* ^27^.

**Table 1.** Demographic characteristics of learners enrolled in government- funded high schools in Khayelitsha, Soweto and Tembisa Townships, NSSF by sex.

Characteristic	Overall (1034)	Female (n= 547)	Male (n=487)	Total (n=1034)
	% (n)	% (n)	% (n)	% (n)
***Sex***				
Female	52.9 (547)	-	-	-
Male	47.1 (487)			
Total	100.0 (1034)			
***Age** (mean=16; SD=1.36)*				
13–15 years	38.5 (398)	61.1 (243)	38.9 (155)	100.0 (389)
16–18 years	58.2 (602)	49.0 (295)	51.0 (307)	100.0 (602
>=19 years	3.3 (34)	26.5 (9)	73.5 (25)	100.0 (34)
Total	100.0 (1034)	52.9 (547)	47.1 (487)	100.0 (1034)
***Race***				
African	100.0 (1034)	52.9 (547)	47.1 (487)	100.0 (1034)
Total	100.0 (1034)	52.9 (547)	47.1 (487)	100.0 (1034)
***Site***				
Khayelitsha	14.4 (149)	61.1 (91)	38.9 (58)	100.0 (149)
Soweto	47.3 (489)	52.6 (257)	47.4 (232)	100.0 (489)
Tembisa	38.3 (396)	50.2 (199)	49.7 (197)	100.0 (396)
Total	100.0 (1034)	52.9 (547)	47.1 (487)	100.0 (1034)
***Grade***				
Grade 9	37.1 (384)	55.2 (212)	44.8 (172)	100.0 (384)
Grade 10	35.5 (367)	48.5 (178)	51.5 (189)	100.0 (367)
Grade 11	27.4 (283)	55.5 (157)	44.5 (126)	100.0 (283)
Total	100.0 (1034)	52.9 (547)	47.1 (487)	100.0 (1034)

**Table 2.** Experience of violence and safety in government-funded high schools in South Africa, by sex.

Variable	Overall	Females	Males	Total	P-value	95% Conf. Interval
	% (n)	% (n)	% (n)	% (n)		
**Physical violence: Experience of Peer provocation and relational aggression**					**0.001**	**1.20-2.07**
Yes	29.1 (301)	44.8 (135)	55.1 (166)	100.0 (301)		
No	70.9 (733)	56.2 (412)	43.8 (321)	100.0 (733)		
**Total**	**100.0 (1034)**	**52.9 (547)**	**47.1 (487)**	**100.0 (1034)**		
**Physical violence: Experience of Peer violence perpetration and victimisation**					0.620	**0.19-5.53**
Every day or most days this term	3.3 (34)	58.8 (20)	41.2 (14)	100.0 (34)		
Once a month this term	2.7 (28)	50.0 (14)	50.0 (14)	100.0 (28)		
Once a week this term	3.2 (33)	45.4 (15)	54.5 (18)	100.0 (33)		
Once this term	7.2 (75)	46.7 (35)	53.3 (40)	100.0 (75)		
This has not happened	83.6 (864)	53.6 (463)	46.4 (401)	100.0 (864)		
**Total**	**100.0 (1034)**	**52.9 (547)**	**47.1 (487)**	**100.0 (1034)**		
**Sexual violence: Peer-perpetrated sexual harassment**					0.564	**0.63-1.28**
Yes	14.2 (147)	55.1 (81)	44.9 (66)	100.0 (147)		
No	85.8 (887)	52.5 (466)	47.5 (421)	100.0 (887)		
**Total**	**100.0 (1034(**	**52.9 (547)**	**47.1 (487)**	**100.0 (1034)**		
**Safety: Perception of feeling unsafe to and from school**					**0.003**	**0.54-0.88**
Yes	48.2 (498)	57.6 (287)	42.4 (211)	100.0 (498)		
No	51.8 (536)	48.5 (260)	51.5 (276)	100.0 (536)		
**Total**	**100.0 (1034)**	**52.9 (547)**	**47.1 (487)**	**100.0 (1034)**		
**Bullying and verbal abuse: Experience of identity-based bullying**					0.305	**0.16-11.0**
Every day or most days this term	5.2 (54)	57.4 (31)	42.6 (23)	100.0 (54)		
Once a month this term	2.3 (24)	54.2 (13)	45.8 (11)	100.0 (24)		
Once a week this term	1.5 (16)	50.0 (8)	50.0 (8)	100.0 (16)		
Once this term	5.4 (56)	66.1 (37)	33.9 (19)	100.0 (56)		
This has not happened	85.5 (884)	51.8 (458)	48.2 (426)	100.0 (884)		
**Total**	**100.0 (1034)**	**52.9 (547)**	**47.1 (487)**	**100.0 (1034)**		
**Bullying and verbal abuse: Experienced verbal abuse from a peer or educator**					0.680	**0.10-5.03**
Every day or most days this term	12.2 (126)	50.8 (64)	49.2 (62)	100.0 (126)		
Once a month this term	6.1 (63)	54.0 (34)	46.0 (29)	100.0 (63)		
Once a week this term	8.1 (84)	47.6 (40)	52.4 (44)	100.0 (84)		
Once this term	16.6 (172)	50.6 (87)	49.4 (85)	100.0 (172)		
This has not happened	57.0 (589)	54.7 (322)	45.3 (267)	100.0 (589)		
**Total**	**100.0 (1034)**	**52.9 (547)**	**47.1 (487)**	**100.0 (1034)**		
**Drugs and alcohol: Exposure to illicit drugs on school property**					0.726	**0.72-1.59**
Yes	89.2 (922)	52.7 (486)	47.3 (436)	100.0 (922)		
No	10.8 (112)	54.5 (61)	45.5 (51)	100.0 (112)		
**Total**	**100.0 (1034)**	**52.9 (547)**	**47.1 (487)**	**100.0 (1034)**		
**Effectiveness of School Management on learner safety: Enforcement of discipline ** **at school**					**0.002**	**0.47-0.85**
Yes	76.8 (794)	55.5 (441)	44.5 (353)	100.0 (794)		
No	23.2 (240)	44.2 (106)	55.8 (134)	100.0 (240)		
**Total**	**1000 (1034)**	**52.9 (547)**	**47.1 (487)**	**100.0 (1034)**		
**Effectiveness of School Management on learner safety: Ability to comfortably report any ** **form of violence to educators**					**0.001**	**0.45-0.74**
Yes	58.3 (603)	58.5 (353)	41.5 (250)	100.0 (603)		
No	41.7 (431)	45.0 (194)	55.0 (237)	100.0 (431)		
**Total**	**100.0 (1034)**	**52.9 (547)**	**47.1 (487)**	**100.0 (1034)**		
**Effectiveness of School policies: Presence of code of conduct pertaining to various forms ** **of violence at school**					0.363	**0.65-1.17**
Yes	78.2 (809)	53.6 (434)	46.3 (375)	100.0 (809)		
No	21.8 (225)	50.2 (113)	49.8 (112)	100.0 (225)		
**Total**	**100.0 (1034)**	**52.9 (547)**	**47.1 (487)**	**100.0 (1034)**		
**Effectiveness of school policies: Exposure to life skills lessons at school**					0.151	**0.65-1.07**
Yes	51.6 (534)	55.1 (294)	44.9 (240)	100.0 (534)		
No	48.4 (500)	50.6 (253)	49.4 (247)	100.0 (500)		
**Total**	**100.0 (1034)**	**52.9 (547)**	**47.1 (487)**	**100.0 (1034)**		

### Experiences of violence and safety, by sex

Table 2 describes experiences of violence and safety in public high schools in South Africa, by sex and the 11 composite variables. Overall, almost 30% (29.1%, n=301) have experienced peer provocation and relational aggression: 7.2% (n=75) who reported that they experienced peer violence perpetration and victimisation once in the school term while 83.6% (n=864) had not experienced this type of violence at all. Fourteen percent (n=147) have experienced peer perpetrated sexual harassment and almost 50% (48.2%, n=498) who reported that they had a perception of feeling unsafe to and from school. More than 5% (n=56) reported that they had experienced identity-based bullying and 16.6% (n=172) experienced verbal abuse from a peer or educator once in the school term. While almost 90% of the participants (89.2%, n=922) were exposed to illicit drugs on the school property, 76.8% (n=794) reported that discipline was enforced in their schools. Over half of participants (58.3%, n=603) reported that they were able to comfortably report any form of violence to educators, and 78.2% (n=809) indicated that there was a code of conduct that pertained to various forms of violence at school. Half (51.6%, n=534) reported being exposed to life skills lessons at school.

The results show that there is a statistically significant association between 4 of the 11 composite variables in relation to sex: experience of peer provocation and relational aggression, perception of feeling unsafe to and from school, effectiveness of school management on learner safety (particular focus on enforcement of discipline at school) and ability to comfortably report any form of violence to educators and sex (p-value<0.05).

When looking at the differences in violence between females and males, over half (55%) of male learners have experienced peer provocation and relational aggression compared to 45% of female learners (p<0.001). Almost 60% of female learners (58%) reported feeling unsafe on their way to and from school, compared to 42% of male learners (p<0.003). Over half (55%) of male learners reported that their school was not effective in enforcing discipline relating to any form of violence: this contrasts with 55% of females noting the opposite (p<0.002). Most females (58%) noted the ability to comfortably report any form of violence to educators, compared to 44% of males (p<0.001).

Whilst the following do not show statistically significant sex differences, these findings are important to note. Female learners represent the largest proportion of those that experience peer violence perpetration and victimisation most days a term: 59% females vs 41% males. Over half (55%) of females and 44% of males have experienced peer perpetrated sexual harassment. Fifty-three percent (53%) of female learners and 47% of males had exposure to illicit drugs on the school property. Only half (54%) of female learners noted the presence of a code of conduct pertaining to various forms of violence at school: this was only 46% for male learners. Less than half of male learners (45%) reported exposure to life skills lessons at school: this rose to 55% for female learners.

## Discussion

Overall, we found high rates of violence among 1034 learners in the 15 government-funded South African high schools who participated in this research. Almost 30% experienced peer provocation and relational aggression, 14% experienced peer perpetrated sexual harassment and almost 50% reported they felt unsafe to and from school and almost 90% were exposed to illicit drugs on the school property. Despite this, 76.8% indicated that discipline was enforced in their schools and 58.3% felt able to comfortably report any form of violence to educators.

We found sex differences in experiences of safety at public high schools with males experiencing more peer provocation and relational aggression at school while female learners were more concerned with safety threats in the community, on their way to and from school. The role of the School Management team in ensuring safety and enforcement of discipline was viewed differently between male and female learners, with females feeling more comfortable to report any form of violence to educators. Our overall findings regarding learner experiences of violence are consistent with other research findings and reports, reinforcing that school safety remains a concern in SA schools ^2, 20, 38, 39^.

Our findings showed four key sex differences in experiences of violence, which will be outlined in turn.

Our findings indicate that male learners are more susceptible to peer provocation and relational aggression compared to females. This is confirmed by existing literature recognising men’s vulnerability to violence ^40^ especially perpetrated by male strangers or acquaintances ^41, 42^. Constructs of masculinity and femininity that position men as dominant and highly sexually active and women as subordinate and acquiescent have been found to contribute towards gender inequality and in turn, to violence ^43^. Interventions such as the SASA! intervention could be implemented, through community mobilization, tackling the social and cultural norms to address the primary prevention of GBV and HIV ^44^. The SASA! intervention was associated with lower past-year of experience of physical and sexual intimate partner violence among women, highlighting that addressing social and gender norms can result in reduction of violence ^44^.

Community safety was a concern for female learners. This is confirmed by literature that indicates that community crime and violence is a concern in SA ^45^, highlighting the need for a more ecological approach to safety, including community safety with an inter-governmental approach. Various evidence-based interventions could be deployed such as the Walking Bus Project ^46^, self-defence workshops ^47, 48^ and the development of community safety plans through Community Safety forums ^49^, with the South African Police Service (SAPS) playing an active role in mapping and ensuring safety.

The poor enforcement of discipline relating to any violence at school experienced by male learners, correlates with and is confirmed by the high rates of peer provocation and relational aggression experienced at a school level. We suggest that learners are involved in the enforcement of discipline as well as strengthening the code of conduct to allow for a more ‘whole school approach’ to addressing this issue, rather than top down. Strategies for restorative discipline should be implemented ^50^, encouraging cycles of peace, rather than punitive discipline, which is currently the norm ^51^.

We found that females felt more comfortable reporting violence to educators, than their male counterparts. More research could be done to better understand why males are not comfortable reporting and also to explore if this finding translated to an increase in reporting of violence at a school level by females. However, with the recent case of a Limpopo learner committing suicide after being bullied ^52^, schools need to re-think and strengthen the anti-bullying policy intervention strategies at all levels ^53^ to identify more subtle types of violence, like bullying, correctly and swiftly. The introduction of anonymous reporting systems could also help encourage reporting of all types of violence. In addition, interventions, like the GAP Year intervention ^26^, should be done at a school level to educate and empower boys on the reporting pathway in a school environment, increasing their ability and confidence to report whilst reducing the stigma. As with the other interventions noted, their success will depend on the extent to which all stakeholders, including learners, educators and other school staff, as well as parents, and the wider community are committed to reducing bullying ^54^.

While not statistically significant, our findings on sexual harassment and exposure to illicit drugs are concerning, however they do align with other studies. Our results confirm findings from Kutywayo *et al.* ^55^ and Ward *et al.* ^16^ indicating that both female and male learners are vulnerable to sexual harassment and violence. The Optimus Study school survey ^16^, and other South African studies ^2, 56^ also found similar results: one in 10 of young people had experienced unwanted sexual touching by a known or unknown adult in their lifetime ^21^. Campaigns and interventions, supported by the government ^57^, should be promoted to normalise the reporting of all types of violence and ensure the adequate first-line support to providers to respond to disclosures of violence. First-line support using the LIVES framework ^2^, provides practical care and responds to a survivors’ emotional, physical, safety, and support needs ^58^.

Given that in 2001, all South African schools were declared drug-free zones and no person may possess illegal drugs on school premises ^59^, it is concerning that almost 90% of all learners had exposure to illicit drugs on the school property. These concerns are confirmed by other studies ^60^. As with the other interventions noted in this manuscript, an ecological approach is critical to address the root cause of this drug problem and break the cycle. The SAPS, the South African National Council on Alcoholism and Drug Dependence (SANCA) ^3^ and the Substance Abuse Prevention through Academic Excellence program ^61^, provide potential interventions.

Several of these findings suggest there is an urgent need for the DBE to strengthen the functionality of the School Safety Committees (SSC), a substructure of the School Governing Body. The participation of the SAPS in the SSC’s is also critical as they conduct random checks and ensure safety from gang related violence, among other safety prevention methods. The recent digitisation of the NSSF and the Protocol on the Management of Sexual Abuse and Harassment training ^62^, outlining the roles and responsibilities of the SSC, is one step in creating a more functional SSC. All the interventions mentioned in this manuscript should be led by the SSC, in collaboration with the other stakeholders. One opportunity for future research would be to explore the variables that create an exceptional school.

### Limitations

Data collection relied upon learner recall of experiences which may result in an under-reporting of violence ^63^. There was a larger sample size of females than males which may have skewed the data. We only gathered data from 15 schools across the 3 sites and therefore survey findings are not generalisable to the wider areas of Soweto, Thembisa and Khayelitsha. The 15 schools who did participate were perhaps not reflective of the sites we surveyed as there is a possible participation bias and an over representation in one of the sites. There was also a number of participants with missing data for specific variables: given that the PCA technique was employed to create composite outcomes for each variable of interest and the main objective was to make statistical inferences using a complete dataset and standardized the sample size for our observations of interest, missing data were excluded as it reduced the statistical power of the study resulting in biased results and invalid conclusions. Due to the nature of the study design, response rates were not available: this should be acknowledged as a limitation. Also, given the number of missing values that the dataset entailed, the missing data would have reduced the true representation of the sampled population which could have threatened the validity of the results obtained in the study, and thus result in invalid conclusions. Despite these limitations, the findings provide important insight into NSSF implementation and areas which require strengthening.

### Opportunities to strengthen the administration of the NSSF survey tool and implemenation and recommendations for the NSSF learner tool

Following the use of the NSSF learner tool, we have noted a few limitations and recommendations for adaptation. Question amendments: currently, there is only ‘female’ and ‘male’ as sex options in the tool: we suggest this is amended to include ‘other’ as a sex category to ensure the tool is gender inclusive. When asking about violence, the tool does not ask the sex of the perpetrator and therefore cannot provide transformative interventions to address violence in schools: we suggest adding a field to gather the sex of the perpetrator of violence. Length of the survey tool: The reductive process undertaken in this analysis has allowed us to focus on fewer but relevant questions pertaining to safety and violence. As such we recommend shortening the survey tool, to only include a few questions for each key variable, capturing the most important summary scores for the outcomes of interest. This may also potentially increase the response rate and improve data quality by reducing multicollinearity errors. Content of the tool: The presentation of many variables (similar variables) may provide the same information which may appear to be significant but ultimately provide information that cannot be used to inform future interventions. The use of such a statistical technique adopted in this manuscript may thus assist in summarising the variance explained by each key variable that measures the main construct e.g., physical violence and also helps merge similar constructs together. COVID-19 adaptations: since the outbreak of the COVID-19 pandemic, we suggest the inclusion of various infection control protocols to be included in the NSSF tool. This will support in creating an enabling school environment.

## Conclusion

Findings confirm that school safety and violence continue to be a concern in SA schools. Whilst these behaviours are displayed at school, violence is determined by a range of often inseparable dynamics located at individual, relationship, community, and societal levels ^64^. This strongly indicates that interventions must work across the various ecological levels to effectively see an impact on violence prevention and reduction. There is also a need to strengthen NSSF implementation to address the concerns that were reported by learners to ensure more effective and efficient learner and educator safety within the school environment. School safety can be strengthened through development and implementation of sustainable action plans, including effective incident reporting system for learners and staff, ensuring that referrals and psychosocial support is provided. We further recommend the adaptation of the NSSF learner survey to increase its utility and implementation thereby allowing it to generate more accurate and timely data for the identification of appropriate safety interventions.

## Figures and Tables

**Appendix 1.** Set of questions combined to create outcomes based on Cronbach Alpha score.

Outcomes	Composite variable	Questions included	Alpha score	Overall Scale reliability coefficient/ Cronbach alpha
**Physical Violence**	(1) ***Experience of peer provocation *** ***and relational aggression***	• Have you been injured by a dangerous object on the way to or from school:		**0.71**
		(i) after being bullied;	0.33	
		(ii) because of insults, swearing or hate speech;	0.46	
		(iii) because someone said something bad about my mother, father, or people important to me;	0.52	
		(iv) to protect someone else who was being hurt in some way; because someone was trying to hurt me in some way	0.40	
	(2) ***Experience of peer violence *** ***perpetration and victimization***	• I have been hit, kicked, pinched or punched by a learner;	0.69	**0.78**
		• I have hit, kicked, pinched or punched a learner	0.67	
**Sexual violence**	(3) ***Experience of sexual*** ***harassment from educators or*** ***peers***	• A learner in my grade has touched me sexually or on my private parts without my permission or forced me to touch his / her private parts;		**0.68**
		• a learner in my grade has called me rude, sexual names;	0.64	
		• a learner in a higher grade has called me rude, sexual names;	0.6	
		• an educator has touched me sexually on my private parts or forced me to touch his / her private parts;	0.48	
		• An educator has called me rude, sexual names		
**Feelings of safety**	(4) ***Perception of feeling unsafe to *** ***and from school***	• Are you afraid that someone may threaten or attack you on your way to school?	0.55	**0.70**
		• When you are at school, do you worry about getting home safely?	0.58	
		• Do you feel you need to protect yourself on your way to school?	0.56	
**Bullying and ** **verbal abuse**	(5) ***Experience of identity-based *** ***bullying***	• I have been bullied because of poverty;	0.30	
		• I have been bullied because of my learning problems;	0.42	
		• I have been bullied because of my appearance;	0.40	
		• I have been bullied because of the way I dress;	0.38	**0.83**
		• I have been bullied because of my race;	0.40	
		• I have been bullied because of my culture and religion;	0.40	
		• I have been bullied because of my gender;	0.49	
		• I have been bullied because of my sexual orientation	0.49	**0.71**
	(6) ***Experience of verbal abuse *** ***from educators or peers***	• An educator has sworn at me;	0.45	
		• an educator has shouted at me;	0.51	
		• an educator has insulted or used hate speech with me;	0.43	
		• a learner has sworn at me;	0.38	
		• a learner has insulted or used hate speech with me	0.37	**0.77**
**Exposure to ** **drugs and alcohol**	(7) ***Exposure to illicit drugs on *** ***school property***	• Have you seen learners smoking cigarettes on the school property?	0.67	**0.68**
		• Have you seen illegal drugs on the school property	0.68	
**Effectiveness** ** of school ** **management**	(8) ***Enforcement of discipline at *** ***school***	• Do learners who use dangerous objects in your school get disciplined?	0.54	**0.70**
		• learners who use illegal drugs at school are disciplined;	0.53	
		• learners who bully others are disciplined at our school;	0.49	
		• learners who fight or physically hurt others get disciplined.	0.38	
**Effectiveness ** **of school ** **management**	(9) ***Ability to comfortably report *** ***any form of violence to educators***	• We can tell the educator if there is discrimination	0.32	**0.87**
		• We can tell the principal if educators discriminate against us	0.67	
**Effectiveness of ** **school policies**	(10) ***Presence of code of conduct *** ***pertaining to various forms of *** ***violence at school***	• We are taught about the dangers of drugs and alcohol;	0.33	**0.70**
		• we have rules about dangerous objects at school;	0.38	
		• we have rules about alcohol and drugs at our school;	0.46	
		• we have rules about bullying at our school	0.41	
		• we have rules about verbal violence at our school;	0.4	
		• we have rules about physical violence and fighting at our school;	0.34	
		• we have rules about sexual violence at our school	0.52	
	(11) ***Exposure to life skills lessons *** ***at school***	• We are taught life skills lessons about discrimination, tolerance and diversity;	0.45	**0.70**
		• We are taught life skills lessons about sexual violence and what to do if we are victims	0.56	

## Data Availability

Harvard Dataverse: National School Safety Framework (NSSF) learner survey data. https://doi.org/10.7910/DVN/PGHJES ^27^. This project contains the following underlying data: GAPYear_NSSF_Learner_Data.tab (survey responses). Harvard Dataverse: National School Safety Framework (NSSF) learner survey data. https://doi.org/10.7910/DVN/PGHJES ^27^. This project contains the following extended data: GAPYear_NSSF_Learner_Survey.pdf (survey tool). Data are available under the terms of the Creative Commons Zero "No rights reserved" data waiver (CC0 1.0 Public domain dedication).
