# Supplementary material for: Learner experiences of safety at public high schools in three South African townships: Baseline findings from the National School Safety Framework learner surveys
Source: Gates Open Res. 2024 Feb 19;6:6. Originally published 2022 Feb 7. [Version 3] doi: 10.12688/gatesopenres.13328.3 (PMC10739271; doi:10.12688/gatesopenres.13328.3)
Supplement: Peer review history

# Reviewer report: Reviewer response for version 3

**DOI:** 10.21956/gatesopenres.16717.r35956

**Published:** 2024-02-29

**Authors:** Evi Widowati

**Affiliations:** 1 Universitas Negeri Semarang, Semarang, Central Java, Indonesia

In my opinion, this article has been revised very well according to the input given by the reviewer, aspects of improvement have been revised in detail, especially in the chapters on methods, sampling techniques, how to select samples, ethical aspects in research and practical suggestions given, thank you.

Is the work clearly and accurately presented and does it cite the current literature?

Yes

If applicable, is the statistical analysis and its interpretation appropriate?

Partly

Are all the source data underlying the results available to ensure full reproducibility?

Partly

Is the study design appropriate and is the work technically sound?

Partly

Are the conclusions drawn adequately supported by the results?

Partly

Are sufficient details of methods and analysis provided to allow replication by others?

Partly

Reviewer Expertise:

Public health, occupational safety and health, child safety education, school safety, child protection, and gender equality.

I confirm that I have read this submission and believe that I have an appropriate level of expertise to confirm that it is of an acceptable scientific standard.

# Reviewer report: Reviewer response for version 2

**DOI:** 10.21956/gatesopenres.16277.r35656

**Published:** 2023-12-21

**Authors:** Evi Widowati

**Affiliations:** 1 Universitas Negeri Semarang, Semarang, Central Java, Indonesia

- Please explain how to select samples from grades 9-11 to be included in your research. Are they all involved?

 - Do students fill out informed consent before they are involved in collecting your data?

 - What do you do on the questionnaire if you may find that data was filled in incompletely during the survey, considering that you collected no personal data?

 - It is recommended that the presentation of the results in the table and discussion should include the results of perceptions and experiences relating to the eight safety domains as the focus of this research, presented in more detail so that readers can follow them better. Apart from that, the discussion chapter should be discussed by comparing relevant points in several other countries (could be causes, best practices or others) considering that this is an international journal with an international readership.

 - It would be more useful if the practical advice given in the conclusion chapter could be given in detail and operationally, including covering who should do it and what.

 - It may be necessary to consider that ethical approval is not only needed when taking the GAP but also regarding your ethics in conducting this research, including treating the data you have obtained.

Is the work clearly and accurately presented and does it cite the current literature?

Yes

If applicable, is the statistical analysis and its interpretation appropriate?

Partly

Are all the source data underlying the results available to ensure full reproducibility?

Partly

Is the study design appropriate and is the work technically sound?

Partly

Are the conclusions drawn adequately supported by the results?

Partly

Are sufficient details of methods and analysis provided to allow replication by others?

Partly

Reviewer Expertise:

Public health, occupational safety and health, child safety education, school safety, child protection, and gender equality.

I confirm that I have read this submission and believe that I have an appropriate level of expertise to confirm that it is of an acceptable scientific standard, however I have significant reservations, as outlined above.

# Response

**Published:** 2024-02-09

**Authors:** Alison Kutywayo

**Affiliations:** Wits Reproductive Health and HIV Institute, South Africa

Thank you for these comments to strengthen our manuscript. 

 - Regarding the sample selection, the survey excluded grade 8 and grade 12 learners; grade 8 learners were new and less familiar to the school environment while grade 12 learners would have left the school when endline assessments were conducted in the following year, as per DBE directive. Classes were randomly selected from participating grades, ensuring at least 1 class per grade was selected, per school. This detail is already noted in the manuscript. 

 - Informed consent: As detailed in the manuscript, the NSSF is a nationally mandated program of the Department of Basic Education, and these data were collected on behalf of the Department of Basic Education. The learner survey is part of routine Department of Basic Education programming and not a research study. In line with this, no learner consent forms were completed – however, learner assent was verbally provided, with willing learners completing the survey.  

 - Incomplete surveys: Once completed, surveys were reviewed for data completeness. However, there were still missing values. These were excluded to yield results only for those who had responded to all the key variables. 

 - Presentation of the results: Given the large size of this dataset, with 152 variables to be analysed, the authors needed to streamline the number of variables for the manuscript. Therefore, we used the principal component analysis, to reduce the number of variables to those that were statistically highly correlated, allowing us to capture strong data patterns in these eight domains. This is well explained in the manuscript already. 

 - Discussion to refer to literature from other countries: We have added a few sentences in the Discussion, referring to a review of school safety interventions and interventions recommended in different countries.  

 - Further detail in Conclusion: we have added in more granular details to outline who is responsible for these conclusions.

# Reviewer report: Reviewer response for version 2

**DOI:** 10.21956/gatesopenres.16277.r35388

**Published:** 2023-11-27

**Authors:** Beverly Kingston

**Affiliations:** 1 Center for the Study and Prevention of Violence, University of Colorado Boulder, Boulder, CO, USA

I have reviewed the article and approve the article for indexing. I noticed a few places that could still be written a little stronger but they are minor editorial issues.

 Here are a couple:

- Last sentence of first paragraph about bullying was vague.
- Table 2 describes experiences of violence and safety in public high schools in South Africa, **overall and** by sex **for** the 11 composite variables. (I added the words highlighted in bold.)

Is the work clearly and accurately presented and does it cite the current literature?

Partly

If applicable, is the statistical analysis and its interpretation appropriate?

Partly

Are all the source data underlying the results available to ensure full reproducibility?

Yes

Is the study design appropriate and is the work technically sound?

Partly

Are the conclusions drawn adequately supported by the results?

Yes

Are sufficient details of methods and analysis provided to allow replication by others?

Partly

Reviewer Expertise:

NA

I confirm that I have read this submission and believe that I have an appropriate level of expertise to confirm that it is of an acceptable scientific standard.

# Response

**Published:** 2024-02-09

**Authors:** Alison Kutywayo

**Affiliations:** Wits Reproductive Health and HIV Institute, South Africa

Thank you for these comments. The sentence about bullying has been removed and we have amended the title of Table 2, as per your guidance.

# Reviewer report: Reviewer response for version 1

**DOI:** 10.21956/gatesopenres.14571.r33899

**Published:** 2023-07-28

**Authors:** Heather Meyer Reynolds

**Affiliations:** 1 Department of Teacher Education, SUNY Empire State College, Saratoga Springs, New York, USA

This paper seems to be covering a gap in the research - which is the routine monitoring of school safety. Monitoring is an important first step to make decisions about prevention and intervention.

 Table 3 has some interesting data but presenting this data as a percentage of 100 for each question is a little bit confusing. It would be much more interesting to see what percent of the whole population surveyed said, "every day" vs. "once a month". I noticed this was also true of other tables. It is just a little confusing.

 Discussion provides some interesting preventive programming and suggestions for future research.

Is the work clearly and accurately presented and does it cite the current literature?

Yes

If applicable, is the statistical analysis and its interpretation appropriate?

Partly

Are all the source data underlying the results available to ensure full reproducibility?

Yes

Is the study design appropriate and is the work technically sound?

Yes

Are the conclusions drawn adequately supported by the results?

Yes

Are sufficient details of methods and analysis provided to allow replication by others?

Yes

Reviewer Expertise:

Creating welcoming and safe school communities.

I confirm that I have read this submission and believe that I have an appropriate level of expertise to confirm that it is of an acceptable scientific standard, however I have significant reservations, as outlined above.

# Response

**Published:** 2023-10-11

**Authors:** Alison Kutywayo

**Affiliations:** Wits Reproductive Health and HIV Institute, South Africa

Thank you for the review of this manuscript. We appreciate your feedback. 

 We have amended Table 1 and 2, (previously labelled 2 and 3) to ensure they now include an ''overall'' column. The descriptive write up (Results) now includes the overall sample size before presenting sex differences.

# Reviewer report: Reviewer response for version 1

**DOI:** 10.21956/gatesopenres.14571.r33697

**Published:** 2023-06-28

**Authors:** Beverly Kingston

**Affiliations:** 1 Center for the Study and Prevention of Violence, University of Colorado Boulder, Boulder, CO, USA

**Some editing is needed to clearly present the work and to ensure the literature review is current:**

- Add population to VAC survey description.
- Add sentence about relationship between substance use and violence.
- There are also characteristics that lead some youth to be bullied more than others; need to mention this in the introduction and integrate into study design.

Copyedits needed; there were some others – article should do a careful review of grammar etc.

 First sentence 2 ^nd^ paragraph – edit to only use enabling once – could say supportive instead.

 Sentence – drug and alcohol – add use;

 principals should not be capitalized;

 Discussion – says relation aggression – change to relational.

**Study Design:**

- It would be helpful for the authors to first present the results of the overall experiences of violence and then show the differences by sex. The levels of violence overall are relevant and important to describe first. By comparing only on sex differences this paper loses the significance of the violence that is affecting both genders.
- This paper would be much stronger if it also looked at the relationship between discrimination and bullying and violence (e.g., were those that were discriminated against more likely to be bullied?)
- Response rates are not available so the survey could be biased; this was acknowledged by authors, but it should also be put it into the limitation section
- Add alpha scores to Table 1.

**Discussion:**

- Add overall discussion of findings before sex breakdown.
- In the discussion section the authors could recommend:
  Conducting focus groups to better understand why males are not comfortable reporting concerning behavior;
  Education about how to be an upstander;
  Implementation of anonymous reporting systems can also help encourage reporting; these kind of strategies can help address all forms of violence (e.g., sexual harassment; illicit drug use; suicide).

Is the work clearly and accurately presented and does it cite the current literature?

Partly

If applicable, is the statistical analysis and its interpretation appropriate?

Partly

Are all the source data underlying the results available to ensure full reproducibility?

Yes

Is the study design appropriate and is the work technically sound?

Partly

Are the conclusions drawn adequately supported by the results?

Yes

Are sufficient details of methods and analysis provided to allow replication by others?

Partly

Reviewer Expertise:

Violence prevention; school safety

I confirm that I have read this submission and believe that I have an appropriate level of expertise to confirm that it is of an acceptable scientific standard, however I have significant reservations, as outlined above.

# Response

**Published:** 2023-10-11

**Authors:** Alison Kutywayo

**Affiliations:** Wits Reproductive Health and HIV Institute, South Africa

Thank you for the review and feedback on this manuscript. 

 We have amended the manuscript to include your recommendations for strengthening: references have been checked, however, there is a lack of current literature therefore the literature appears old. Copyedits have been made. We have amended Table 1 to include the ''overall' results before describing the results by sex: this change has also been made to the Results. You suggested a few recommendations for the Discussion - these have been incorporated.
